# Impact of the second wave of COVID-19 pandemic on food security among Ho indigenous community of Jharkhand, India

**DOI:** 10.1186/s40066-024-00469-1

**Published:** 2024-04-15

**Authors:** Suparna Ghosh-Jerath, Ayushi Dhasmana, Swati C. Nair, Ridhima Kapoor

**Affiliations:** 1 The George Institute for Global Health, Delhi, India

**Keywords:** Tribal, Ho community, Second wave, COVID-19, Food security, Food consumption

## Abstract

**Background:**

Food insecurity and hunger are global concerns further exacerbated by the unprecedented COVID-19 pandemic. There is a need to understand the depth of this impact, especially among smallholder farmers, and recognize specific coping strategies that offered resilience to inform preparedness in future. The present cross-sectional study assessed the impact of the second wave of COVID-19 pandemic on different dimensions of food security among smallholder farmers of Ho indigenous community of Jharkhand, India. It also explored potential resilient attributes of their food systems.

**Results:**

Most of the respondents (67.2%) reported reduced food consumption at the household (HH) level. Majority faced difficulty in accessing food from different food sources; however, easier access to government food security programmes was highlighted. Around 40% reported change in their ability to purchase farming inputs. Market vendors reported disruptions in food procurement owing to travel restrictions; however, no change was reported for home-produced foods in agricultural lands/kitchen gardens. Prices of indigenous foods produced locally decreased/remained same; however, for cereals, pulses, and other HH staples, prices increased during second wave. Difficulty in accessing wild food environment (OR: 1.7, CI 0.40, 7.75), change in food prices (OR: 19.9, CI 5.25, 76.02), decrease in HH income (OR: 9.2, CI 2.99, 28.60) were found to be significantly associated with reduction in HH food consumption (*p* < 0.01). The coping strategies adopted by the community included sale of cultivated and wild produce in local weekly markets to ensure additional income.

**Conclusions:**

The findings highlight the need to reinforce the traditional ecological knowledge of the Ho community and focus on practices around their food systems, engrained into their socio-cultural ecosystems that may offer resilience against future stresses. In addition, the need of systemic support to ensure the social and economic well-being of the community needs to be prioritized.

## Background

Prior to the onset of COVID-19 pandemic, Food and Agriculture Organization identified conflicts and climate vulnerabilities as major drivers of food insecurity and global hunger [1]. While these challenges persist, the COVID-19 pandemic and its cascading impact on different facets of the food environment have been rampant. According to FAO estimates, between 720 to 811 million people faced global hunger in 2020 which was about 181 million more than the prevalence reported in 2019 [1]. Furthermore, about 2.37 billion people across the world could not access adequate food in 2020 (increased by 320 million people in just 1 year), and around 50% of these people were from Asia, facing moderate to severe forms of food insecurity [1]. In this way, the COVID-19 pandemic and its social and economic consequences have dramatically changed the trajectory towards achieving Sustainable Development Goals (SDGs). The pandemic has also severely impacted 26 of the 230 commitment goals (established in 2013 and 2017 Nutrition for Growth summit) towards reducing malnutrition, which can be attributed to the lack of funding and/or diversion of national revenue and resources towards COVID-19 management and mitigation [2].

Similar to the global fallout, food security among the Indian population was also affected as a direct impact of COVID-19 pandemic on livelihood and the resultant economic slowdowns. In India, agriculture, and allied sectors are the largest source of livelihoods, employing about 45.6% of total population in the workforce (in 2019–20) [3] and contributing to 20.2% (in 2020–21) of the total national income [4]. Virus containment measures such as border closure, and restrictions in trading and transport impeded the farmers from timely producing and harvesting their crops. This was directly influenced by the shortage of labour and difficulty in accessing markets, including purchase of farm inputs and equipment. These changes limited their productive capacities and hindered the sale of their produce [5–7]. It also disrupted the local food supply chains and reduced access to healthy, safe and diverse diets among the masses [8]. Literature has highlighted loss of income and livelihood along with the disruption in access to health and nutrition services among the Indian population as a direct impact of the COVID-19 pandemic [9, 10]. Although agriculture sector survived the first wave, propagation of second wave was more rapid and brought newer challenges for the farmers, who were already under pressure due to price volatility and rising debts [11, 12]

About 70% of Indian rural households (HH) primarily rely on agriculture for their livelihood and about 82% of these are marginal and smallholder farmers [13]. The second wave of the pandemic in the country and the following virus containment measures came at a time when the rabi (winter) crop was ready for harvest. Among the smallholder farmers of Jharkhand, Bihar, Odisha and Andhra Pradesh, the second wave noticeably impacted access to equipment for harvesting and caused labour shortages. In addition, transport restrictions and temporary closure of markets hindered the sale of their agricultural yield [11]. In addition, while experiencing income losses, these smallholder farmers also resorted to coping mechanisms, such as distress sales, taking out additional loans, and engaging in child labour [6]. All these impacts of COVID-19 lockdowns has been notable among the indigenous smallholder farmers, impacting their overall food systems [11, 14].

Around 8.6% of the total population in India belongs to various indigenous communities, identified as scheduled tribes (STs) [15, 16]. Jharkhand, an eastern Indian state, known for its rich biodiverse agroforestry [17] is home to several indigenous communities that constitute 26.2% of the state’s population [15]. Jharkhand is home to 32 indigenous communities, constituting 8.6 million population in the state, out of which 0.9 million of the population (10.7%) belong to Ho Indigenous community [18]. The Ho community is a predominantly smallholder farmer community, who mainly earn their livelihood from agriculture and sale of wild produce [19]. Limited literature is available to understand the impact of COVID-19 pandemic on food security of Ho community. Our previous work on Santhal, Munda and Sauria Paharia indigenous communities of Jharkhand [14] showed that despite the impact of COVID-19 pandemic on food accessibility, availability and consumption, the smallholder farmers demonstrated unique attributes of resilience concerning their food systems. Some of the notable resilience attributes included their ability to access diverse natural food environments, dependence on indigenous seeds, and involvement of family members in farming practices during the lockdown. Improved access to fair price shops when local informal markets experienced shocks to food supply and shifts in prices also helped in minimising the impact of the pandemic [14]. Similar findings were also reported among Ho tribe, wherein the respondents had sufficient locally available indigenous foods and were concerned about inability to sell produce and livestock at local markets [20].

While most of the evidence highlight the disruption of livelihood and services to be more profound during the first wave of the pandemic, there is a need to understand the impact of the second wave on the food systems of vulnerable indigenous smallholder farmers. It is imperative to identify their resilient attributes and utilise them to develop disaster-preparedness strategies and effectively mitigate these situations in future. In the current study, we explored the impact of the second wave of COVID-19 pandemic on different dimensions of food security among the smallholder farmers of Ho indigenous community of Jharkhand, India. We specifically explored how they utilized various types of food environments when food systems were already disrupted due to the mani-fold effects of the first wave. In addition, we explored the impacts on farming practices, market prices and food consumption at the HH level. Our study also explored the potential resilient attributes concerning the food systems of the Ho community. These findings could be utilized to build stronger and resilient food systems for a nutritionally secure future, both locally and globally.

## Methodology

### Study design

A cross-sectional HH survey was conducted in September 2022 to understand the impact of second wave of COVID-19 pandemic on the food systems of Ho indigenous community. The respondents were asked retrospectively about the impact they faced during April to June 2021. This was the time period when Jharkhand state experienced the second wave of COVID-19, which led to spiralling cases, increased deaths and consequently, state-imposed lockdowns. In addition, a market survey was also conducted to explore the impact on informal weekly markets.

### Study area and population

This study was part of a larger project that examined indigenous food consumption by indigenous communities of Jharkhand and its contribution to dietary diversity and food security among women and children [21].

**Fig. 1:**
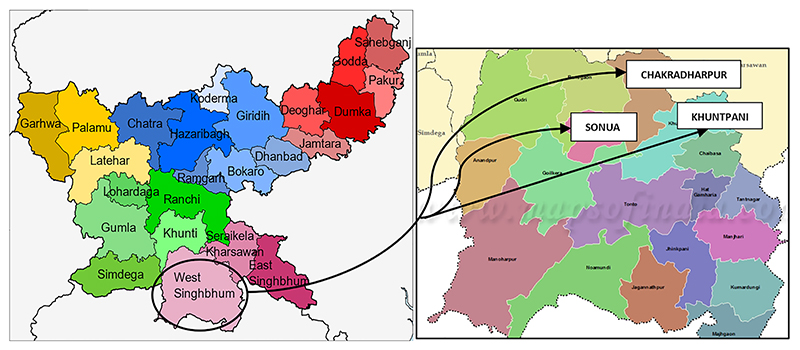
Selection of Chakradharpur, Sonua, and Khuntpani blocks from West Singhbhum district of Jharkhand, India

The West Singhbhum district of Jharkhand, with a total area of 10,863 Sq. Km and 15 blocks, has total population of about 15 lakh, out of which 7.7 lakh are members of Ho tribe [18, 22]. Three geographically diverse blocks with high population of Ho indigenous community were purposively selected, namely, Sonua, Khuntpani and Chakradharpur (Fig. 1). The COVID-19 survey was conducted at the HH level, wherein the data were collected from any adult member of the HH who agreed to participate.

### Sample size calculation

The sample size for the COVID-19 survey was calculated using the formula for estimation of proportion, considering the prevalence of reduced HH income among smallholder indigenous farmers in Jharkhand during the pandemic as 77% [14]. With 95% confidence interval and taking precision as 7% while adjusting for 5% non-response, the final sample size estimated was 211. In the current study, data from 213 respondents were captured and analyzed.

### Sampling strategy

Based on the larger objective of the project, a two-stage cluster sampling design was followed. At the first stage, ten villages were randomly selected from the purposively chosen blocks (Sonua (1), Khuntpani (5), and Chakradharpur (4)) using probability proportional to size (PPS) sampling. Secondly, a house listing exercise was carried out in 1036 HHs for constructing the sampling frame of eligible HHs for the larger study. The COVID survey was conducted in every fourth household to obtain the requisite sample size. For the market surveys, a total of ten informal weekly markets that were accessed by the Ho indigenous community from the study villages were chosen.

### Study tools

**Fig. 2:**
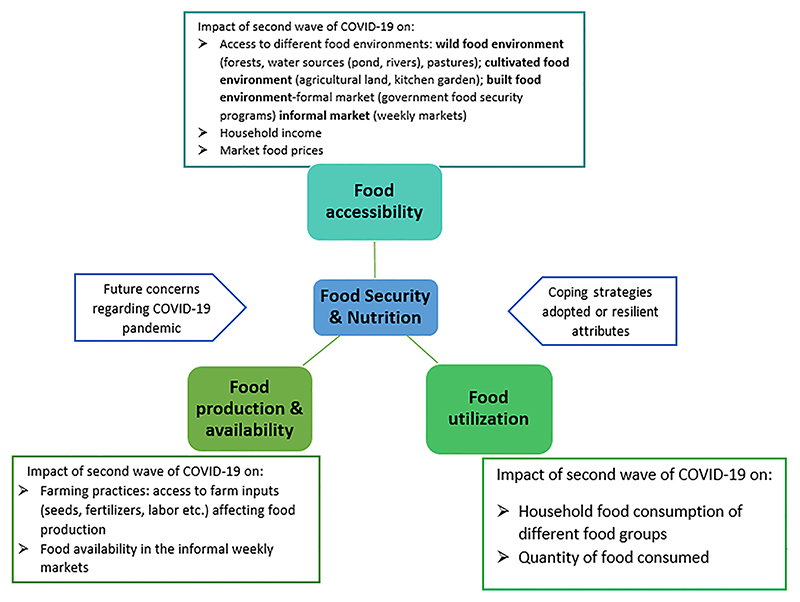
Variables assessed as part of the COVID-19 survey to understand the impact of second wave of COVID-19 pandemic on different dimensions of food and nutrition security

A modified and adapted version of tool “COVID-19 Surveillance Community Action Network (C-SCAN)” [23] based on the food environment typology framework [24], was used. The tool was modified to specifically capture the impact of second wave of the pandemic on different dimensions of food and nutrition security among the Ho indigenous community (Fig. 2). The survey elicited information regarding the socio-demographic profile, along with the perceptions of community regarding the impact of second wave of COVID-19 pandemic on food production and availability in informal weekly markets, access to different food environments, and food utilization. In addition, it also captured information on specific resilient attributes, and perceptions around pandemic induced future concerns. The survey tool was administered by the local field investigators from the Ho community who received training from the core team.

The market survey tool was adapted from a tool administered to examine markets in Myanmar [25] and was administered to the food vendors by the local field investigators in the markets accessed by the study villages. This tool elicited information on the following parameters: (i) main types of foods sold in the market, (ii) food prices during pre-COVID times and second wave, along with perceived reasons for change, (iii) sources of food procurement and any change in procurement patterns with the reason, and (iv) change in sale of food items along with perceived reasons. A set of additional questions were administered to the food vendors (one vendor from each market), which elicited their perceptions on changes in sales and income due to the second wave of COVID-19 pandemic.

### Data collection and data entry

The field investigators administered the survey tool on an electronic data capture platform using Samsung tablets (Model SM-T385). The C-SCAN survey tool was incorporated in CS-Pro Software, Version 7.6, which provided in-built checks (range, context, and logic checks) for maintaining data quality. The market survey tool was administered in paper forms by the field investigators. For use in a local context, both the study tools were administered in Hindi to facilitate the communication of core team members with the indigenous communities, who mainly understood the Hindi language.

### Data analysis

The data from CS-pro software was exported, cleaned, and coded in MS excel and the analysis was performed in Stata version 15.1. The data were analysed in terms of percentages with frequencies for categorical variables and mean with standard deviation for continuous variables. This study explored the association of reduction in quantity of food consumption with various independent variables, such as age, gender, change in the access of various food sources, food prices, farming practices and HH income, with bivariate analysis using Chi square or Fisher exact test. All the variables that showed significant association (*p* < 0.05) were transferred to the binary logistic regression model [26] to explore their association with outcome as reduction in quantity of HH food consumption. Given the dichotomous nature of dependent variable, simple adjusted model without any modifications was applied after checking all the assumptions.

## Results

The following sections report the socio-demographic profile of the Ho indigenous community and impact of second wave of COVID-19 pandemic on various dimensions of food security, such as food production and availability, accessibility, and utilization. Further-more, it explores the factors associated with the change in HH food consumption. Finally, this paper explores the specific coping strategies adopted by the community that offered resilience during the pandemic.

Out of the total 213 respondents surveyed, a majority were males (73.3%), and the mean age of the respondents was 40.5 ± 14.3 years. Details on the profile of the respondents and their HHs is provided in Table 1.

### Impact of second wave of COVID-19 pandemic on different aspects of HH food security

#### Food production

The people belonging to Ho indigenous community are dependent predominantly on agriculture-based livelihoods [19]. Major part of their food baskets comprises home-grown foods produced in the cultivated food environment consisting of agricultural lands and kitchen gardens. Therefore, we explored the impact of COVID-19 on their farming practices (Table 2). Majority of respondents (80.8%) did not change their farming practice during the second wave. Out of those who reported a change (*n* = 41 HHs), the common changes included involving family members for farm labor (*n* = 40/41 HHs), more use of natural fertilizers, such as cow dung (*n* = 36/41 HHs) and more use of indigenous seeds (*n* = 33/41 HHs). This can be attributed to availability of family members (due to reverse-migration) and limited access to markets to purchase hybrid seeds, chemical fertilizers and other farm inputs.

Around 40% of the respondents reported disruption in access to farming tools and the commonly reported disruptions were in ability to purchase hybrid seeds (*n* = 74/85 HHs) and chemical fertilizers (*n* = 71/85 HHs) during the pandemic times.

#### Food availability in informal weekly markets

In local markets (*n* = 10) catering to the selected study villages, the vendors reported procuring foods locally within the district or village level. In addition, they also reported procuring foods from neighbouring districts and states for mainly hybrid varieties of pulses, other vegetables, roots and tubers, flesh foods, vegetable oil, spices, and packaged foods. During the second wave, disruptions in food procurement were reported in the local markets owing to lockdown (*n* = 7), transport restrictions (*n* = 4), non-availability of some seasonal vegetables as they were utilized for HH consumption or reduced production (*n* = 5). However, some vendors reported no change in the procurement of mainly (though not limited to) indigenous varieties of rice (*n* = 2), pulses (*n* = 6), green leafy vegetables (*n* = 3), other vegetables (*n* = 8), roots and tubers (*n* = 6), and flesh foods (*n* = 6). This was attributed to sale of HH produce from the agricultural lands, kitchen gardens and HH rearing of livestock, in the local markets.

#### Food accessibility

Almost all the respondents reported accessing the wild food environment (96.3%), such as forests, ponds/rivers (water sources), and pastures, and cultivated food environment (97.2%), such as agricultural land and kitchen garden. In addition, the respondents also reported accessing foods from built food environment comprising formal markets (government food security programs) and informal weekly markets. Table 3 reports the changes in the food environment of the community due to the second wave of COVID-19 pandemic.

More than half of the respondents reported facing difficulties in accessing foods from cultivated food environment (63.4%), during the second wave as compared to pre-COVID time. This was attributed to reduced ability to purchase farm inputs which led to difficulty in food production in cultivated lands. Furthermore, due to travel restrictions in lockdown, 53% respondents (mainly from study villages situated far from the forest areas) reported reduced access to wild food environment. Though majority of respondents (74.2%) reported a change in receiving food from various government schemes, this change varied for different food security schemes. For Targeted Public Distribution System (TPDS), majority of the respondents (72.8%) reported easier access, whereas for supplementary food provision under Integrated child development services (ICDS), a majority reported difficulty in access to hot cooked meals (53.8%) and take-home rations (43.1%). More than half of the respondents reported difficulty in accessing food from Mid-day meal scheme (56.4%).

In addition to these schemes, majority of the respondents (69.5%) reported provision of food under newly launched schemes. This included receiving additional free ration (5 kg of rice/wheat and 1 kg of pulses per person per month in a household), cash transfer from *Pradhan Mantri Garib Kalyan Yojna* (PM-GKAY) and dry ration (rice) in lieu of Mid-day meal scheme. Others (2.6%) reported receiving benefits of cooked meal/free ration from non-governmental organizations (NGOs), such as Integrated Development Foundation (IDF) and Tribal Research and Training Center (TRTC).

About 89.7% of the respondents reported hardships in accessing food from informal markets during the second wave. Along with an overall reduction in food access from different sources, reduction in HH income (78.8%) was also reported. The common reasons for reduction in income included restricted migration (76.2%), lack of work opportunities (79.8%), relatively lower wages (75.6%), low sale of agricultural produce (55.9%) and low sale of food products for market vendors (51.8%). Decrease in HH income was found to be significantly associated with difficulty in access to market foods (*p* < 0.05).

### Local food prices

**Fig. 3:**
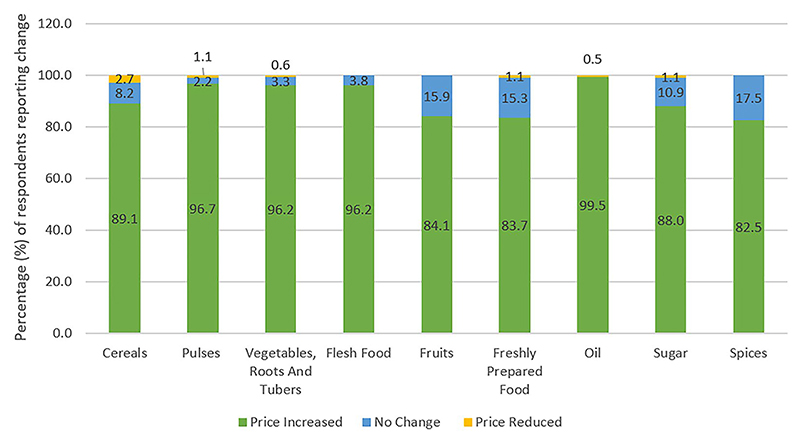
Change in price of various food groups during the second wave of COVID-19 pandemic as compared to pre-COVID times

Change in market food prices were reported by majority of the respondents (85.9%), with more than 80% of them reporting price inflation for all food groups (Fig. 3). Almost all (99.5%) of the respondents reported an increase in price of cooking oils.

**Fig. 4:**
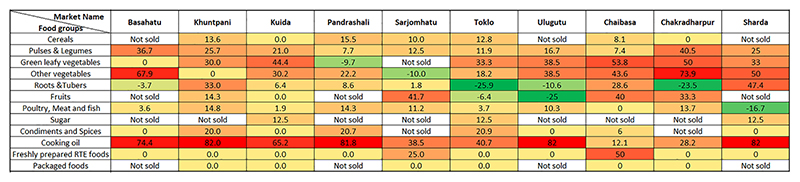
Heat map on impact of second wave of COVID-19 pandemic on the retail prices of the food groups in the informal markets in Ho indigenous community. *RTE* Ready to eat. Green shading represents the food groups that were sold at lower prices during the second wave of COVID-19 pandemic as compared to the pre-pandemic times with darker green shade representing higher percentage decrease in price, whereas the red shading represents the food groups that were sold at higher prices with darker red colour representing higher percentage increase in price. Yellow shading represents no change

In the market surveys, increased prices were reported for cereals, pulses, flesh foods, sugar, spices, cooking oil and freshly prepared processed foods in all the markets (Fig. 4). The main reasons reported for increase in price of cereals and pulses were, rise in wholesale prices and increased transportation charges. Some markets (*n* = 4) also reported that they increased the price of cereals and pulses due to the ongoing price inflation. In most markets (*n* = 6), no price change was observed for indigenous varieties of pulses such as horse gram, red gram, green gram, and black gram as vendors reported production of these indigenous pulses in their agricultural fields or kitchen gardens. In most markets, increase in prices of green leafy vegetables (*n* = 7), other vegetables (*n* = 8) and roots and tubers (*n* = 6) were observed, due to reduced production, lower market availability and increase in wholesale prices. Contrary to this, indigenous vegetables such as cowpea, *tupi leaves*, colocasia leaves, kovai, and ashgourd were being sold at cheaper prices in some of the markets (*n* = 4). The common reasons reported were production in kitchen gardens and subsequent decrease in wholesale prices of these indigenous foods. Highest percentage increase in the range of 65–82% was observed in the price of cooking oil in 6 out of 10 markets due to increase in the wholesale price. All the markets reported reduced sales of foods owing to low availability of certain foods and temporary closure of the markets due to lockdown measures. However, vendors in some markets (*n* = 4) reported increased sales of certain foods, such as potato (due to lower price as compared to other vegetables), mixed vegetable spices (lower price as compared to other spices), fish and pork (used as an alternative for chicken as people were apprehensive about contracting COVID-19 from chicken).

### Food utilization (household food consumption)

**Fig. 5:**
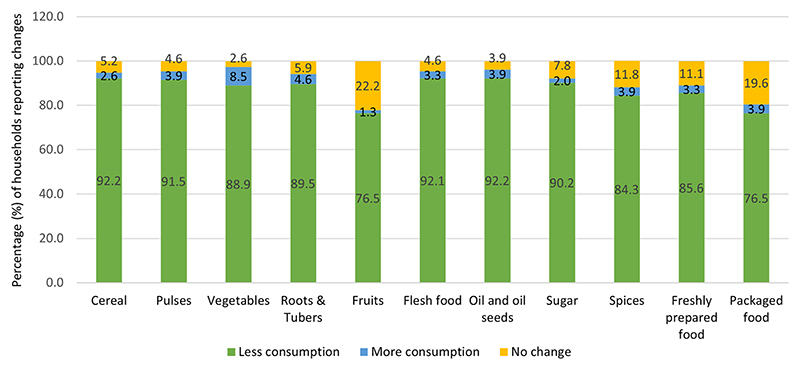
Change in HH food group consumption during the second wave of COVID as compared to pre-COVID times

Table 4 reports the change in HH food consumption during the second wave of COVID-19. A change in consumption of various food groups was reported by majority of HHs (71.8%). Around 67.2% reported that they consumed food in lesser quantities than pre-COVID times. This was attributed to increase in food prices, reduced income, and closure of local markets. More than 90% of the HHs reported reduced consumption of cereal, pulses, flesh food, sugar along with cooking oil (Fig. 5).

*Factors associated with change in HH food consumption* Difficulties in accessing food from cultivated food environment (agricultural land, kitchen gardens), wild food environment (forest, water bodies, pastures), and built food environment including both formal markets (government programs) and informal weekly markets were significantly associated with reduced HH food consumption (*p* < 0.05). In addition, change in farming practices, food prices, and decrease in HH income were also found to be significantly associated with reduced HH food consumption (*p* < 0.05).

The variables that showed significant association in the bivariate analysis (*p* < 0.05), such as access to different food sources, change in food price, change in farming practices and decrease in HH income, were transferred to the multivariate logistic regression model for adjusted analysis. It was found that HHs with difficulty in accessing food from wild food environment were 1.7 times (*p* < 0.01, CI 0.40, 7.75) more likely to decrease their food consumption as compared to those who had easier access to the same. In addition, change in food prices and HH income also had a significant impact on food consumption. HHs that reported a decrease in income were 9.2 times (*p* < 0.01, CI 2.99, 28.60) more likely to decrease their food consumption.

As compared to the HHs that reported no change in food price, those who reported a change were 19.9 times (*p* < 0.001, CI 5.25, 76.02) more likely to decrease their food consumption (Table 5).

### Future concerns regarding COVID-19 and coping strategies

Around 60% of respondents expressed their concern about HH food security owing to future impact of COVID-19 pandemic. The major concerns relating to HH food security in future included non-availability of food (90.6%), difficulty in food affordability (95.6%), and difficulty in food consumption (90.6%).

The coping strategies during the second wave suggested by the community included selling their cultivated (12%) and wild produce (7.5%) in the local weekly markets to earn additional income. Common varieties of foods sold included green leafy vegetables, rice and other vegetables from farms and wild edible mushrooms and green leafy vegetables from forests and open pastures (Table 6).

## Discussion

The smallholder farmers belonging to Ho indigenous community of West Singhbhum district of Jharkhand demonstrated changes in their food security status during the second wave of COVID-19 pandemic. Changes were observed in procurement of farm inputs, food access from natural food environment and informal markets and HH food consumption. Disruptions were reported in procurement of most food items by market vendors during the lockdown period. Difficulty in access to wild food environment, change in food prices, and decrease in HH income had significant impact on reduced HH food consumption. Resilient attributes towards impact of COVID-19 on food security included easier access to government food security schemes, mainly TPDS and sale of cultivated and wild produce in local weekly markets for additional income. The respondents faced challenges in accessing the farm inputs during the second wave of pandemic. Similar challenges were reported from farming communities of Andhra Pradesh, Bihar, Jharkhand and Odisha, wherein inability to purchase farming equipment, fertilizers/pesticides, unavailability of labor, and lack of storage/warehouses were the key concerns [11, 27]. In addition, some studies from India and abroad have reported these factors as a direct fallout of first wave of COVID-19 among the rural farming communities [7, 28–31]. Among smallholder farmers, these challenges may hamper their production capacity. There is evidence from literature which emphasizes on measures, such as crop insurance, providing subsidies for agricultural inputs, providing assistance in terms of agricultural technology, and cash transfers as effective modalities to mitigate such challenges [32, 33]. Systemic support to promote shorter supply chains is also one way to ensure easier and direct sales by farmers as well as easier access to consumers [34].

The market closure and its impact on access to food and dietary diversity have been reported in literature from rural parts of India [35]. This was also evident in the developed countries, where the HH food basket pre-dominantly comprises market foods (resulting in a surge in demand from supermarkets) [31, 36]. Contrary to this, in the rural communities of Jharkhand, the dependence on the natural food environment along with the market offered a resilient attribute, whereby the impact of the COVID-19 pandemic was somewhat reduced owing to the access to food from their natural environment [14]. In the current study, half of the respondents reported facing difficulties in accessing their natural food environment. Therefore, it becomes imperative to provide information, education, and communication around effectively accessing biodiverse food sources. This can be an important coping strategy for communities having rich traditional ecological knowledge and residing in the vicinity of the natural food environment [14]. Evidence based on case studies from countries such as Hawaii, Australia, Brazil and some Asian countries have shown that encouraging hile-gardens/home gardens can ensure a strong local food economy hile also safeguarding the nutritional requirements of the population [37]. The findings from around the world highlight that utilizing the natural food environment and using the traditional knowledge of indigenous communities not only encourages a diverse food basket but is also a sustainable technique as it encourages local and shorter supply chains. A systematic review on the impact of COVID-19 on food and nutrition security in LMICs also reported that farming communities with shorter value chains were better placed to survive the pandemic [38]. These shorter procurement chains can reduce the damage inflicted on both consumers and farmers owing to the sudden restriction in movements thereby ensuring economic as well as food and nutrition security [34]. This was an observation in our study also, wherein the vendors that procured foods at the village level or sold their own HH produce did not report any difficulty in procurement of these foods.

The formal food market catered by the fair price shops worked effectively and in fact were more accessible during the second wave of the pandemic than the pre-pandemic times among the Ho indigenous community. This was a critical observation in some reports that TPDS served as an important safety net for ensuring food security during the COVID-19 crisis when the regular markets/food supply chains became dysfunctional [39, 40]. While this is an encouraging trend, better accountability, and control mechanisms, and reducing transportation cost through supply chain optimization, may further help to strengthen these food security programs. Additionally, strengthening the existing grievance redressal mechanisms, monitoring through social audits, and transparency through automating the entire supply chain may result in better access under these programs [40, 41].

The respondents reported reduction in their HH income which was significantly associated with decrease in HH food consumption. Similar finding was reported from other farming communities, wherein about 50% of farmers stated that reduction in HH income during the second wave made it difficult to purchase quantity of foods similar to pre-pandemic times [11]. This high-lights the need for creating economic security which is an important determinant for ensuring household food security. Thus, creating an enabling environment, wherein in addition to promoting traditional practices for food and nutrition security, fostering increased access (particularly for youth) to agricultural advisory services, improving labour productivity through skill training, setting up rural-based small-scale agro-industries and promoting integrated farming systems may provide increased employment opportunities [42, 43]. This may result in young, smallholder farmers to continue to stay in their own communities. The uptake of Mahatma Gandhi National Rural Employment Guarantee Act (MGN-REGA), a wage-for-employment policy of Government of India went down during the pandemic due to lower wages and payment delays [44]. Measures for continuity and massive expansion of such programs are needed to deal with high work demand, especially for vulnerable communities who are the key beneficiaries and their access to such programs are reflected in their nutritional status [45].

## Conclusion

The present study provides crucial observations on the impact of second wave of COVID-19 on perceived food security in hard-to-reach indigenous community of India, The Ho community has demonstrated some resilient traits, which to some extent, have helped them cope with the adverse impacts of COVID-19 pandemic on their food security. However, the community requires systemic support to sustain their well-being, especially during adverse situations, such as the pandemic. Our study has highlighted the need to reinforce the traditional ecological knowledge of the Ho community and focus on practices around their food systems, engrained into their socio-cultural ecosystems that may offer resilience against future stresses (such as pandemics). However, there is also a need to economically empower them with effective and targeted programs and policies. Measures such as crop insurance, subsidized agricultural inputs and use of agricultural technology can be used to ensure the social security of these subsistence farmers. Coordinated actions are required across different sectors to provide support to vulnerable indigenous communities and guide them against future threats to their food systems. With the right multi-sectoral support, the Ho indigenous community of Jharkhand, may have the potential to set an example for other vulnerable communities on sustainable use of their biodiverse resources while also taking charge of their nutritional well-being.

### Policy implications

Findings from this study can help policymakers better understand the resilient traits of indigenous communities, which can be harnessed to further enhance their capacity to withstand future threats to food security. Regulatory reforms around investments in technological development, improving the reach of crop insurance schemes, and agricultural subsidies can provide sufficient resources to augment social and economic empowerment in these communities. Thus, such reforms are likely to facilitate government’s effort in fostering productive, sustainable, and resilient food systems. Supporting small-holder farming communities with sufficient resources can help in harboring traits that build their self-sufficiency to fulfil their economic and nutritional needs. The evidence generated from this work highlights the role of rich traditional ecological knowledge and shorter supply chains as attributes towards creating a food secured environment for vulnerable communities during the pandemic. Furthermore, there is a felt need for contextualized and targeted social, economic and agricultural welfare schemes that have the potential to enhance pre-paredness of small-holder farming communities towards future pandemic.

### Study limitations

The findings of the current study need to be interpreted in light of some limitations. First, the current study has a cross-sectional design with inherent flaw of not providing temporal associations. Secondly, since the survey was administered on any consenting adult of the HH, there could be chances that the respondent may or may not be responsible for food production and/or collection which may have influenced our study findings. Finally, we acknowledge that we have used perception-based tool to assess food security scenario, which may have some inherent limitations. However, we are also cognizant that some of the recommended and validated global food security assessment tools also use perception-based approach of assessment. None-theless, the study findings provide crucial information on how the second wave of COVID-19 pandemic has impacted various aspects of food security among Ho community. This tool can further inform future research for developing and validating similar tools to measure impact of any natural disaster or pandemics on various dimensions of food security.

## Figures and Tables

**Table 1:** General profile of surveyed HHs in Ho indigenous community, Jharkhand, India

Characteristic (*N* = 213)	*n* (%)
Age of respondents (in years)	
18–40	116 (54.5)
41–80	97 (45.6)
Gender of head of the HH	
Female	107 (50.3)
Male	106 (49.8)
Gender of respondents	
Male	156 (73.3)
Female	57 (26.8)

**Table 2:** Change in farming practices among Ho indigenous community during the second wave of COVID-19 pandemic as compared to pre-COVID-19 times

S. no	Variable	*n* (%)
1	*Change in farming practices (N =* 213)	41 (19.2)
1.1	Type of change* (*N* = 41)	
	Involving family members for farm labour	40 (97.6)
	More use of natural fertilizers (cow dung)	36 (87.8)
	More use of indigenous seeds	33 (80.5)
	Paying higher farm wages	19 (46.4)
	Started early farming	17 (41.5)
	Delayed farming	18 (43.9)
2	*Change in access to farming tools* (N = 213)	85 (39.9)
2.2	Type of change* (*n* = 85)	
	Ability to buy seeds	74 (87.6)
	Ability to buy manure	71 (83.5)
	Availability of labour	54 (63.5)
	Availability of farm equipment	66 (77.7)

* *Respondents reported multiple options*

**Table 3:** Change in access to different food environment among Ho indigenous community during second wave of COVID-19

S.no	Characteristic	*N* (%)
1	Change in access to food from wild food environment (forests, water sources, pastures)	
	Easy	28 (13.2)
	Difficult	114 (53.5)
	No change	63 (29.5)
	Not applicable	08 (3.8)
2	Change in access to food from cultivated food environment (agricultural land, kitchen garden)	
	Easy	29 (13.6)
	Difficult	135 (63.4)
	No change	43 (20.2)
	Not applicable	06 (2.8)
3	Change in access to food from built food environment—formal market (government food security programs) (*n*= 158)	
3.1	Change in receiving subsidized food items from TPDS^1^	
	Easy	115 (72.8)
	Difficult	28 (17.7)
	No change	11 (7.0)
	Not applicable	04 (2.5)
3.2	Change in receiving Hot cooked meal under ICDS^2^	
	Easy	26 (16.5)
	Difficult	85 (53.8)
	No change	06 (3.8)
	Not applicable	41 (25.9)
3.3	Change in receiving Take home ration under ICDS	
	Easy	35 (22.2)
	Difficult	68 (43.1)
	No change	11 (6.7)
	Not applicable	44 (27.9)
3.4	Change in receiving food from Mid-day meal scheme	
	Easy	28 (17.7)
	Difficult	89 (56.4)
	No change	07 (4.4)
	Not applicable	34 (21.5)
4	Change in access to built food environment—informal weekly markets	
	Easy	08 (3.8)
	Difficult	191 (89.7)
	No change	10 (4.7)
	Not applicable	04 (1.8)

1 *TPDS:* Targeted Public Distribution System provides food grains (10 kg/month/family) to the population falling below poverty line at specially subsidised prices via fixed price shops

2 *ICDS:* Integrated Child Development Services Scheme is a flagship programme by Ministry of women and child development, government of India targeting early childhood care and development

**Table 4:** Change in household food consumption during the second wave of COVID-19 as compared to pre-COVID-19 times

S. no	Variable (n = 213)	*n* (%)
1	Change in HH food consumption (in terms of types of foods consumed)	153 (71.8)
2	Reduction in quantity of food consumed	143 (67.2)
2.1	Reason for less consumption* (*n* = 143)	
	Increase in food prices	140 (97.9)
	Reduced income	139 (97.2)
	Market closure	138 (96.5)
	Reduced access to food from agricultural land and kitchen gardens	100 (69.9)
	Reduced access to food from forests, water sources, pastures etc	59 (41.3)
	Reduced food distribution by the government schemes	37 (25.9)
	Infected with coronavirus	09 (6.3)
	Less availability of food	03 (2.1)
	Future concerns regarding food availability	2 (1.4)

* Respondents reported multiple options

**Table 5:** Factors associated with decrease in HH food consumption in Ho indigenous community, Jharkhand, India

Variable(*N* = 206)	Unadjusted analysis		Adjusted analysis	
	OR (CI)	*p* value	OR (CI)	*p* value
Access to food from cultivated food environment (farm/kitchen garden)				
Easy	Ref	< 0.001	Ref	0.086
Difficult	9.3 (3.8, 22.8)		4.6 (1.17, 18.37)	
No change	1.7 (0.65, 4.73)		2.6 (0.61, 11.53)	
Access to food from wild food environment (forests, pastures, water sources)				
Easy	Ref	**< 0.001**	Ref	0.002*
Difficult	6.5 (2.66, 16.25)		1.7 (0.40, 7.75)	
No change	0.9 (0.40, 2.40)		0.3 (0.07, 1.34)	
Not appli- cable	1.1 (0.23, 5.55)		0.1 (0.01, 1.11)	
Access to food from government schemes				
Yes	Ref	**0.02**	Ref	0.351
No	0.4 (0.25, 0.90)		0.6 (0.23, 1.66)	
Access to food from informal weekly markets				
Easy	Ref	**0.007**	Ref	0.240
Difficult	2.4 (0.59, 10.23)		1.7 (0.21, 14.35)	
No change	0.2 (0.03, 1.99)		0.2 (0.01, 4.87)	
Not applicable	0.3 (0.02, 4.73)		0.4 (0.01, 17.95)	
Change in food price				
No	Ref	**< 0.001**	Ref	< 0.001*
Yes	11.9 (4.58, 30.95)		19.9 (5.25, 76.02)	
Change in farming practices				
Yes	Ref	**0.004**	Ref	0.207
No	0.21 (0.07, 0.57)		0.4 (0.11, 1.51)	
Not appli- cable	0.55 (0.11, 2.67)		1.5 (0.14, 17.20)	
Decrease in HH income				
No	Ref	**< 0.001**	Ref	< 0.001*
Yes	22.4 (9.17, 54.97)		9.2 (2.99, 28.60)	

* Statistically significant at *p* < 0.01

**Table 6:** Future concerns regarding COVID-19 and coping strategies

S. no	Variable	*N* (%)
1	Future concerns about HH food security owing to COVID-19	128 (60.1)
1.1	Types of concerns* (*n* = 128)	
	Difficulty in affordability of food	112 (95.3)
	Difficulty in food consumption	116 (90.6)
	Non-availability of food	116 (90.6)
	Difficulty in food accessibility	115 (89.9)
	Difficulty in farming practices	109 (85.2)
	Reduced income	09 (7.0)
2	Coping strategies	
2.1	Initiated sale of agricultural/kitchen garden produce in informal market	26 (12.2)
2.1.1	Type of food item sold* (*N* = 26)	
	Green leafy vegetables	19 (73.1)
	Rice	15 (57.7)
	Other vegetables	14 (53.9)
	Millets	04 (15.4)
	Pulses	02 (7.7)
2.2	Initiated sale of produce sourced from wild food environment in informal market	16 (7.5)
2.2.1	Type of food item sold* (*N* = 16)	
	Mushrooms	13 (81.3)
	Green leafy vegetables	13 (81.3)
	Other vegetables	10 (62.5)
	Roots and tubers	09 (56.3)
	Fruits	06 (37.5)

* Respondents reported multiple options

## Data Availability

The data sets used and/or analysed during the current study are available from the corresponding author on reasonable request.
